# Enhancing microbial-induced calcium carbonate precipitation efficiency in calcareous sands through ferric ion additives: A comprehensive experimental investigation

**DOI:** 10.1371/journal.pone.0327568

**Published:** 2025-07-09

**Authors:** Jin Zhu, Renjie Wei, Di Dai, Liangliang Li, Zhiyang Shang, Zhao Jiang, Jie Peng

**Affiliations:** 1 School of Civil Engineering, Wanjiang University of Technology, Maanshan, China; 2 Key Laboratory of Ministry of Education for Geomechanics and Embankment Engineering, Hohai University, Nanjing, China; 3 College of Civil Engineering, Yancheng Institute of Technology, Yancheng, China; Independent Researcher, The UK Global Talent Exceptional Promise, UNITED KINGDOM OF GREAT BRITAIN AND NORTHERN IRELAND

## Abstract

In recent years, the reinforcement of calcareous sands using the microbially induced calcium carbonate precipitation (MICP) method has emerged as a prominent research area. Nevertheless, a significant drawback of the MICP method is that multiple treatments with the cementing solution are required to achieve the desired improvement effect. To address this limitation, this study proposes an optimized MICP strategy through adding the ferric ion into cementing solutions. The effectiveness of the proposed method was investigated by analyzing the precipitation of CaCO_3_, unconfined compressive strength (UCS) and permeability coefficient through aqueous solution test and sand column reinforcement test. Experimental results revealed that ferric ion incorporation significantly altered CaCO_3_ crystal morphology and particle size distribution in aqueous solution test. In sand column tests, specimens treated with cementing solution with ferric ion achieved the UCS of 2.83 MPa after five injection cycles, representing a 15-fold increase compared to conventional MICP-treated specimens under the same test conditions. At the same time, permeability coefficients decreased by two orders of magnitude relative to untreated sand. The micro-structure analysis showed that ferric ions were involved in the reaction to generate a clogging precipitate, which changed the distribution of bio-CaCO3 in the pores of the soil, thereby improving the cementation efficiency. These findings indicate that the addition of ferric ion can overcome the shortcoming of frequent treatment of cementing solution in MICP-reinforced calcareous sand, and provide new insights for the development of effective biological grouting strategies.

## Introduction

MICP is a biogeochemical process mediated by bacteria that induces the precipitation of CaCO_3_ by enzymatic hydrolysis of urea in the presence of calcium ions. Since Whiffin’s work [1] demonstrated its potential for sand stabilization, MICP technology has garnered significant attention in geotechnical engineering [2–6]. The underlying mechanism involves urease-driven urea decomposition, yielding CO₃^2^⁻ and NH₄ ⁺ , which subsequently combine with ambient Ca^2^⁺ to form interparticle CaCO_3_ cementation. This biomineralization process effectively reduces soil permeability by pore-filling and enhances mechanical strength through particle bonding [7].

As a bio-mediated soil improvement technique, MICP has achieved wide application in geotechnical engineering. By cementing soil particles and filling pore spaces, MICP demonstrates a variety of applications including soil stabilization [8], seepage prevention [9], liquefaction mitigation [10], and heavy metal remediation in contaminated soils [11]. At the core of the MICP process are urease-producing bacteria, mainly *Sporosarcina pasteurii* in the present study, which promote CaCO_3_ precipitation through urease-catalyzed hydrolysis of urea. The bacterial strain of *Sporosarcina pasteurii* exhibits optimal urease activity at pH 8.0 [12] and temperatures between 20–37°C [13], coupled with remarkable environmental adaptability including survival capability in high-salinity conditions [14]. However, there are still some challenges in applying MICP to practical engineering. Since both the decrease in permeability coefficient and the increase in UCS are directly related to the content of CaCO_3_, and the content of calcium carbonate precipitated in each cycle is limited, the inefficient precipitation efficiency restricts the application of MICP. At low precipitation levels, CaCO_3_ crystal mainly plays the role of pore filling and cannot effectively improve the strength of sand, but can reduce the permeability coefficient of sand to a certain extent [15,16]. When the content of CaCO_3_ is relatively high, calcium carbonate crystals form cementation between particles, which can significantly increase the UCS of sand. However, an increase in the Ca^2+^ concentration in the cementing solution (> 1M) will inhibit the urease activity [17], limiting the amount of CaCO_3_ precipitate obtained in a single treatment. Therefore, multiple grouting cycles are required to obtain the predetermined CaCO_3_ content. This inefficiency emphasizes the necessity of developing an optimized treatment method to improve the mechanical properties of the sandy soil.

Recent advancements in MICP technology have focused on three strategic optimization approaches to enhance cementing efficiency: (1) Regulating bacterial activity via pH/temperature to improve uniformity of CaCO_3_ distribution [18,19]. Cheng et al. [18] pioneered a novel low-pH one-phase injection method, in which bacterial solution and cementing solution can be injected into the soil together by temporarily inhibiting the urease activity through hydrochloric acid, and this method improves the cementing homogeneity. The experimental results showed that the UCS of the soil was significantly increased to 2.5 MPa after 6 treatments. Meanwhile, the ammonia emission was reduced by 90%, which solved the ammonia pollution problem associated with the conventional MICP scheme. (2) Improving the soil matrix through additives. Incorporation of granular or fiber additives into soil matrix has been proven to effectively optimize MICP cementation [20–22]. Zhao et al. [22] investigated the reinforcing effect of bentonite combined with MICP on coarse-grained soil and its reinforcing mechanism. When the bentonite content was 3% (w/w), the maximum UCS of the treated sample was 5.9 MPa, which was four times higher than that of the control without additives. When bentonite-assisted MICP treatment is used, the permeability can be reduced to 1/ 9920 of the untreated sand due to the enhanced bio-clogging effect. (3) Modifying the cementing solution by using chemical additives [23]. Notably, Jiang et al. [24] demonstrated that the addition of hydrochloric acid to the cementing solution during MICP reinforcement of calcareous sand promotes the dissolution of ions in matrix particles and improves the efficiency of biomineralization. Subsequent studies revealed that aluminum ions released from calcareous materials significantly reduce required cementation cycles [25,26], indicating that specific metal ions can affect the MICP reaction.

Although the enhanced cementation effect of aluminum-based flocculants in MICP-reinforced sand soil has been explored, the research on the application of ferric ions – inorganic flocculants with similar structures – in biological cementation is still insufficient. Similar to recent experimental work by Janssen et al. [27], where the influence of grain size and composition on calcite precipitation was demonstrated, this study investigates how chemical additives alter precipitation and reinforcement patterns in calcareous sands. Crucially, prior investigations of Al^3^⁺ -MICP system [25,26] mainly focused on the strength enhancement mechanism and did not report the effect on the reduction of soil permeability, which is a key gap addressed in this study. This study expands the previous research on the flocculant-MICP system by investigating the role of ferric ions in enhancing the reinforcement of calcareous sand. In addition, we explored the influence of ferric ion-generated flocs on the soil permeability coefficient, providing new methods for the field of seepage prevention.

## Materials and methods

### Materials

#### Bacterial suspension and cementing solution.

The bacterial strain employed in this study was *Sporosarcina pasteurii* (ATCC 11859), a ureolytic microorganism extensively utilized in MICP research due to its high urease productivity and environmental resilience [28]. Following standardized cultivation protocols, the bacteria were propagated in a liquid nutrient medium (pH 7.5, 30°C, 150 rpm agitation) to mid-exponential growth phase, yielding a homogeneous bacterial suspension with an optical density (OD_600_) of 1.3 ± 0.2. Urease activity, quantified via electrical conductivity monitoring of urea hydrolysis kinetics, averaged 8.0 ± 0.5 mmol·L^−1^·min^−1^, confirming enzymatic competence for subsequent bio-cementation processes.

The cementing solution is composed of equal molar concentration (1.0 M) of CaCl_2_ and urea, providing raw materials for urease-driven CaCO_3_ precipitation. In order to evaluate the enhancing effect of ferric ions on the biological cementation of calcareous sand, FeCl_3_·6H_2_O was introduced into the cementing solution. Previous pre-experiments have shown that a very small amount of ferric ions can enhance the strength of MICP-reinforced sand. However, when the concentration of ferric ions is too high, the pH of the cementing solution will drop significantly, thereby having a negative effect on bacteria. Therefore, in this paper, we control the concentration of ferric ions within the range of 0.001M to 0.03M.

#### Test sand.

The sand used in this paper is biological calcareous sand from the South China Sea, which is mainly composed of coral and shell fragments. XRF analysis confirmed that the CaCO_3_ content was more than 90%, containing trace alkaline components, including magnesium carbonate. These irregular particles have high internal porosity and morphological heterogeneity.

### Methods

#### Aqueous solution test.

**Fig 1:**
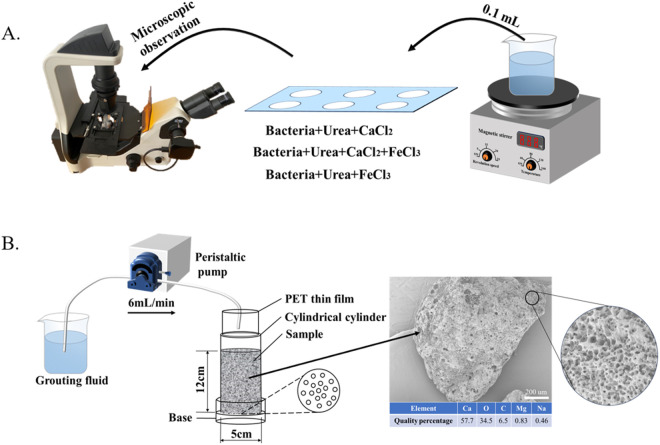
Demonstration of test steps. **A)** Solution test; **B)** Sand column test.

The influence of ferric ions on the precipitation of CaCO_3_ was observed through the aqueous solution test. FeCl_3_·6H_2_O was added to the mixed solution of 1 M CaCl_2_ and 1 M urea, and the concentrations of Fe^3+^ were 0.001, 0.003, 0.005, 0.01, 0.02, and 0.03 M respectively. Meanwhile, two additional experimental groups were set up. One of them did not contain ferric ions, and the solution composition was a mixture of 1 M equal moles of CaCl_2_ and urea, serving as the control group. In the other experimental group, the solution composition was a mixture of 0.02 M ferric ion and 1 M urea. There are a total of eight experimental groups. Take 200 mL of the mixed solution of different groups, and then inject 20 mL of the bacterial suspension. The mixture was stirred continuously for 12 hours using a constant temperature magnetic stirrer (150 rpm, 25 ± 0.5°C), as shown in Fig 1A.

At the initial stage of the aqueous solution, after the mixed solutions of different test groups and the bacterial suspension were mixed, immediately use a pipette to draw 0.1 mL of the liquid into a groove slide with a diameter of 15 mm and a depth of 1 mm. A micro-reaction system was constructed to observe the microscopic morphology of precipitation under a fluorescence microscope. During the reaction, pH meter was used to record the pH change of the solution system. After the reaction, the precipitate was vacuum-filtered (0.22µm membrane) and then dried in an oven at 60 °C for 72 h to obtain the mass of the precipitate.

#### Sand column experiment.

The sand column test device is shown in Fig 1B. Calcareous sand with a particle size of 0.25–0.5 mm was filled in the mold in three layers, each layer being compacted to form a sand column with a bottom diameter of 5 cm and a height of 12 cm. The bottom of the mold has multiple 0.2 mm circular holes to ensure the outflow of the bacterial suspension and the cementing solution. At the beginning of the test, 90 mL (one times the pore volume of the sand column) of the fixing solution was injected into the sand column by a peristaltic pump. The fixing solution was a 0.05 M CaCl_2_ solution, whose main function was to enhance the adsorption of bacteria on the sand [7]. Then, 90 mL of the bacterial suspension was injected. After the injection was completed, the sand column was left to stand for 6 hours to allow the bacteria to be fully adsorbed. Finally, 90 mL of the cementing solution was injected every 12 hours. During the injection process, the injection speed of the peristaltic pump was controlled at 6 mL/min to avoid the reduction of bacterial retention caused by excessive liquid flow rate. The sand column test groups were divided based on the different components of the cementing solution to explore the influence of ferric ions on the MICP reinforcement process of sandy soil. The specific group information is shown in Table 1.

**Table 1:** Grouping of sand column test.

Test Group	Composition of cementing solution	Ferric ion concentration/ M
**A**	Urea+CaCl_2_	0
**B**	Urea+CaCl_2_ + FeCl_3_	0.001
**C**		0.003
**D**		0.005
**E**		0.01
**F**		0.02
**G**		0.03
**H**	Urea+FeCl_3_	0.02

#### The relevant inspection and testing after sand column reinforcement.

After the injection was completed, the samples were taken out of the mold and soaked in deionized water for 24 hours to remove the soluble substances contained in the sand column. Then, the samples were placed in an oven at 60°C for 72 hours to completely dry them for the following tests:

(1) Calcium carbonate content

The amount of CaCO_3_ precipitated was represented by the mass difference before and after the MICP reaction, and the calculation formula is as follows:


$$CCC=\frac{{\text{m}}_{\text{2}}\text{-}{\text{m}}_{\text{1}}}{{\text{m}}_{\text{1}}}\times 100\;\%$$ (1)

Where *m*_*1*_ represents the dry mass of the sand before the reaction, and *m*_*2*_ indicates the mass of the sand column after the reaction.

(2) Permeability Coefficient

The permeability coefficient was directly measured in the sand column test container using a variable head permeability test. The testing and calculation methods refer to ASTM D1557 [29].

(3) UCS

The dried sample was ground into a sand column with a bottom diameter of 5 cm and a height of 10 cm, and placed in an unconfined compression apparatus for mechanical testing (1 mm/min strain rate, ± 0.5% load accuracy) [18].

(4) Microstructure

The SEM test was conducted using a field emission scanning electron microscope. Blocky samples with a particle size of 2 mm were taken for testing. Before the test, they underwent vacuum pre-treatment for 20 seconds, gold spraying for 20 seconds, and then electron microscope scanning.

## Results

### Solution test

The MICP process generates NH₄⁺ and CO₃²⁻ through bacterial-mediated urea hydrolysis. In the presence of Ca² ⁺ , these ions nucleate to form CaCO_3_ crystals, which appear as white spherical particles in the aqueous suspension (Equations 2–5).


$$\text{CO(N}{\text{H}}_{\text{2}}{\text{)}}_{\text{2}}\text{ + }{\text{2H}}_{\text{2}}\text{O}\overset{\text{bacteria}}{\to }\text{ 2N}{\text{H}}_{\text{3}}\text{ + }{\text{H}}_{\text{2}}\text{C}{\text{O}}_{\text{3}},$$ (2)

$$\text{N}{\text{H}}_{\text{3}}\text{ + }{\text{H}}_{\text{2}}\text{O}⟷\text{N}{\text{H}}_{\text{3}}\text{∙}{\text{H}}_{\text{2}}\text{O}⟷\text{N}{\text{H}}_{\text{4}}^{\text{+}}\text{ + O}{\text{H}}^{\text{-}},$$ (3)

$$2\text{O}{\text{H}}^{\text{-}}+{\text{H}}_{\text{2}}\text{C}{\text{O}}_{\text{3}}⟷\text{C}{\text{O}}_{\text{3}}^{\text{2-}}+\text{2}{\text{H}}_{\text{2}}\text{O,}$$ (4)

$${Ca}^{2+}+\text{C}{\text{O}}_{\text{3}}^{\text{2-}}\to Ca{CO}_{3}↓$$ (5)

**Fig 2:**
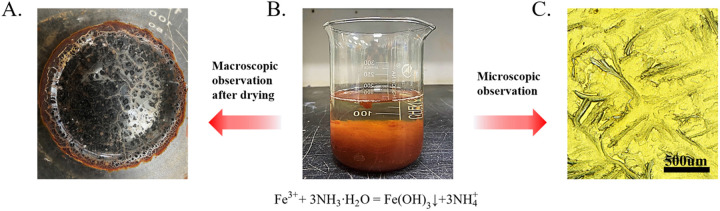
Reaction of Bacteria, Urea and Fe^3^^+^.

The MICP reaction simultaneously generates NH_3_·3H_2_O (Equation 3), increasing the alkalinity of the solution. When ferric ions are introduced into the ammonia water solution, Fe^3^⁺ reacts with OH⁻ to form Fe(OH)_3_, as shown in Fig 2B. The solution contains no Ca^2^⁺, and only the reaction of ferric ions in the solution after bacterial urea decomposition is observed, with a urea concentration of 1 M and a ferric ion concentration of 0.02 M. The experiment observed a large amount of flocculent Fe(OH)_3_ aggregates, which dried to a reddish-brown color and had a mass of 0.65 g, adhering to the surface of the glass beaker (Fig 2A). Fig 2C presents the flocculation observed under a microscope, revealing the formation of dendritic structures.

**Fig 3:**
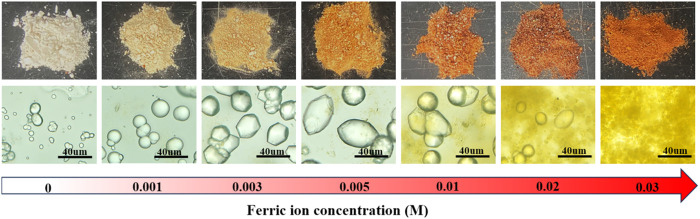
Macroscopic and microscopic morphology of precipitated calcium carbonate.

Fig 3 illustrates the impact of incorporating varying concentrations of ferric ions into the cementing solution on CaCO_3_ formation via MICP. In the control group (no ferric ions), macroscopically, calcium carbonate particles coalesce and appear white. Microscopically, the calcium carbonate exhibits a spherical morphology and is densely aggregated. When ferric ions are introduced into the solution, even at low concentrations, they influence the MICP reaction. As the ferric ion concentration increases, the precipitate's color darkens progressively, eventually adopting a reddish-brown hue. Moreover, the higher the concentration of ferric ions, the more dispersed the overall precipitate. When the ferric ion concentration is greater than 0.02 M, the calcium carbonate particles cannot be effectively adhered to each other.

From a microscopic perspective, at a ferric ion concentration of 0.001 M, the size of calcium carbonate particles is notably larger than in the control group, while maintaining a spherical shape. At 0.003 M, the particle size further increases, and morphological changes begin to occur. At 0.005 M, the CaCO_3_ particles grow larger and adopt an ellipsoidal morphology. At 0.01 M, yellow flocs appear alongside calcium carbonate under the microscope, consistent with the flocculent precipitate observed in Fig 2C. However, in environments where ferric ion concentrations are ≥ 0.01 M, as the ferric ion concentration increases, the size of CaCO_3_ particles decreases. At 0.03 M, granular calcium carbonate is no longer detectable within the microscopic field of view.

**Fig 4:**
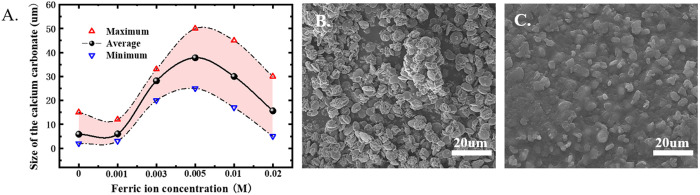
A) Calcium carbonate crystal size versus ferric ion concentration; B) SEM image at ferric ion concentration of 0.03 M; C) Ferric hydroxide colloid.

The size of CaCO_3_ within the microscopic observation range of the microscope was selected as the statistical quantity. The average particle size was calculated, and the maximum and minimum particle sizes were statistically analyzed to obtain the effect of different ferric ion concentrations on the size of CaCO_3_, as shown in Fig 4A. We expressed the effect of ferric ion concentration on CaCO_3_ with a data graph. When the concentration of ferric ion reached 0.03 M, there were no granular CaCO_3_ within the microscopic field of view, so the crystal size was not statistically analyzed in Fig 4A.

To more clearly observe the precipitate in Fig 3 at a ferric ion concentration of 0.03 M, the sample was dried and its morphology was examined under a scanning electron microscope (SEM), as shown in Fig 4B. For comparison, the precipitate in Fig 2A was also analyzed via SEM, as presented in Fig 4C. Observations reveal that the ferric hydroxide in Fig 4C exhibits a colloidal structure, whereas the precipitate in Fig 4B consists of stacked sheet-like structures. This marked difference suggests that the material in Fig 4B remains CaCO_3_, which forms aggregates presenting significant yellow precipitates within the microscopic field of view. This observation aligns with the phenomenon observed when aluminum ions are added to the cementing solution [25]. It is evident that at relatively high concentrations of aluminum or ferric ions, the morphology of calcium carbonate generated by MICP undergoes complete transformation. Based on microscopic observations of the precipitates formed under various conditions, three distinct modes of CaCO_3_ existence can be identified: (1) In the absence of ferric ions or at low ferric ion concentrations, MICP-generated CaCO_3_ exists in a granular form; (2) As the ferric ion concentration increases, yellow flocs begin to form, and further increasing the concentration leads to a decrease in granular CaCO_3_ while generating more yellow precipitates, resulting in a coexistence state of granular and sheet-like forms; (3) At higher ferric ion concentrations, granular calcium carbonate is no longer detectable, and the material transitions entirely into a sheet-like state.

**Fig 5:**
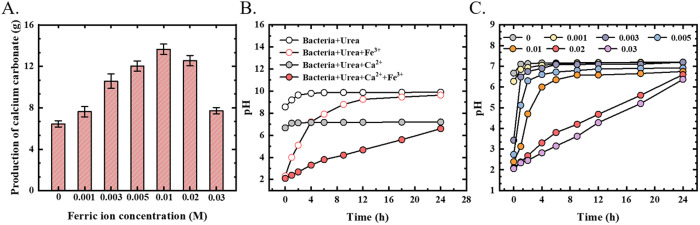
Mass of calcium carbonate produced from aqueous solution and pH change in solution. **A)** Mass of calcium carbonate produced in aqueous solution; B) and C) pH changes in aqueous solution.

Upon completion of the MICP reaction in the aqueous solution, the mass of the generated precipitate was quantified, with the results presented in Fig 5A. When the ferric ion concentration ranged from 0 to 0.01 M, the mass of CaCO_3_ precipitate increased gradually with the increase of ferric ion concentration. However, when the ferric ion concentration surpassed 0.01 M, the mass of CaCO_3_ precipitate began to decrease with further increases in ferric ion concentration. The observed variation in the mass of CaCO_3_ precipitate can be attributed to changes in the pH value of the solution during the reaction process.

The process of urea decomposition by *Sporosarcina pasteurii* increases the pH value in the environment [30] (Equation 2–3). In the presence of Ca^2+^, the CaCO_3_ precipitation reaction is initiated (Equation 4–5), which facilitates the consumption of OH⁻ as described in Equation 4, thereby reducing the final stable pH value, which stabilizes around 7 [31].

Fe^3^⁺ , as a trivalent cation, undergoes significant spontaneous hydrolysis in aqueous solutions. Specifically, hydroxide ions coordinate with Fe^3^⁺ to form hydrolyzed complexes (e.g., Fe(OH)_2_^+^), releasing H⁺ in the process. The release of H⁺ acidifies the solution and consequently lowers its pH value [32]. As shown in Fig 5B, the pH curve variation pattern of the calcium-free experimental group is consistent with that of the calcium-containing group. The addition of Fe^3^ ⁺ significantly lowers the initial pH value of the solution. However, as the MICP reaction progresses over time, the pH value in the Fe^3^ ⁺ -containing solution gradually increases and eventually stabilizes near the final pH equilibrium observed in the solution without Fe^3^⁺ .

There is a competitive pH regulation effect between the simultaneously occurring MICP reaction and the hydrolysis process of Fe^3^⁺ . Studies have shown that the higher the Fe^3^⁺ concentration, the more significant the decrease in solution pH, which inhibits the activity of bacterial urease [33]. Nevertheless, even under acidic conditions, *Sporosarcina pasteurii* demonstrates a certain degree of pH tolerance [18,34,35], thereby maintaining the progress of the MICP process and gradually promoting the recovery of pH through urea decomposition. As illustrated in Fig 5C, an increase in ferric ion concentration not only extends the time required for pH to reach its final stable value but also results in a lower final stable pH value. The change in pH indirectly affects the precipitation efficiency of CaCO_3_ in the MICP solution by influencing the activity of bacterial urease. Lai et al. [31] demonstrated that within the range of bacterial viability and with sufficient calcium availability, moderately reducing the pH can effectively promote CaCO_3_ precipitation. However, when the Fe^3^⁺ concentration exceeds 0.02 M, excessive acidification can cause damage to microbial metabolic activity, leading to a reduction in CaCO_3_ precipitation yield. The MICP reaction and the hydrolysis of Fe^3^⁺ collectively influence the pH changes in the solution, thereby determining the variation pattern of CaCO_3_ precipitation mass in the aqueous solution (Fig 5A).

### Sand column test

**Fig 6:**
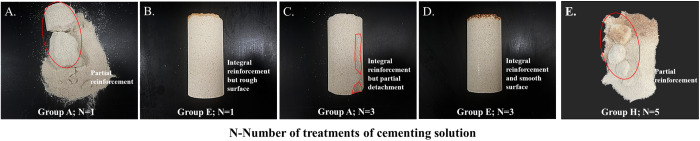
Reinforcement of calcareous sand columns.

The reinforcement effect of the calcareous sand column is presented in Fig 6. Under single-treatment conditions with the cementing solution, the conventional MICP (Group A) formed only localized cementation blocks (Fig 6A), whereas the Fe^3+^-containing experimental group (Group E) achieved overall cementation of the sand column (Fig 6B). After three treatments with the cementing solution, despite surface peeling in Group A, overall cementation was still attained (Fig 6C); Group E presented a smoother surface and an improved cementation level (Fig6D). For the H group without Ca^2+^ ions, after five treatments, effective reinforcement of the sand column could not be achieved. Additionally, due to the interaction between Fe^3+^ and sand particles, localized red aggregates were observed (Fig 6E).

**Fig 7:**
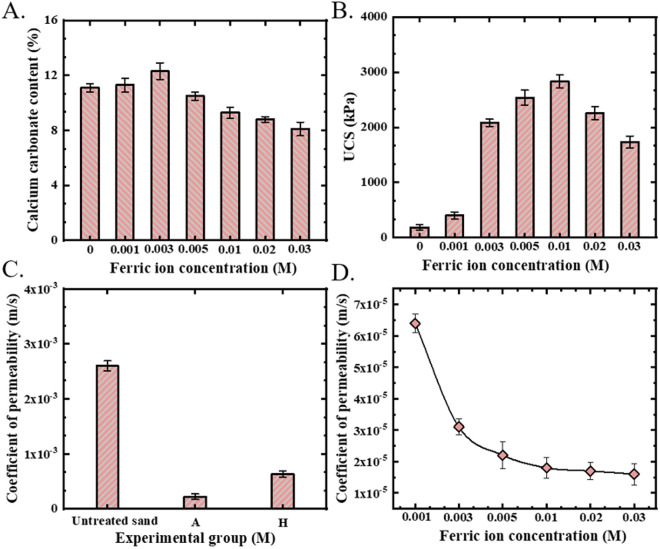
Calcium carbonate content, UCS and permeability coefficient of sand column test. **A)** Calcium carbonate content; **B)** UCS; C) and D) permeability coefficient.

Fig 7 presents the influence of ferric ions on the content of CaCO_3_, UCS, and permeability coefficient. After five treatments with the cementing solution, as the concentration of ferric ions increased from 0.001 M to 0.003 M, the generation of CaCO_3_ gradually increased; however, when the concentration exceeded 0.005 M, the amount of CaCO_3_ decreased, as shown in Fig 7A. Solution tests demonstrated that within a specific concentration range, ferric ions promoted bacterial-induced calcium carbonate formation [31]. Conversely, in the sand column test, this enhancing effect was insignificant. Moreover, when the ferric ion concentration was high, the amount of CaCO_3_ formed in the sand column was lower than that in the test group without ferric ions. This was because in the solution test, the calcium source was abundant, and under such conditions, a lower pH environment would promote more CaCO_3_ precipitation. In contrast, in the sand column test, the calcium ions retained in the pore space of the sand column were restricted. With the increase in the number of injections, the volume of the sand pore space decreased, and less calcium source was left in the pore space available for the MICP reaction. Under these circumstances, the lower pH influenced the precipitation of CaCO_3_ [36].

As shown in Fig 7B, the UCS of each sand column group after five treatments with the cementing solution are presented. The UCS of the conventional MICP test group was 185.5 kPa. With increasing ferric ion concentration, the UCS of the sand columns improved. When the ferric ion concentration reached 0.01 M, the UCS increased to 2832.6 kPa. Comparative analysis revealed that the strengthening mechanisms of Fe^3^⁺ and Al^3^⁺ in the bio-cementation system are similar, enhancing the reinforcement strength of the sand column through particle bonding and pore filling.

MICP effectively reduces soil permeability through CaCO_3_ crystal formation in interstitial pore spaces [37]. In this study, the permeability coefficient of the untreated sand was 2.6 × 10 ⁻ ^3^ m/s. After five treatments with MICP, it decreased by one order of magnitude to 2.2 × 10 ⁻ ⁴ m/s. When the cementing solution consisted of ferric ions and urea, the permeability coefficient of calcareous sand was 6.3 × 10 ⁻ ⁴ m/s after five treatment cycles. This value was also reduced to some degree compared to that of the untreated sand, indicating that ferric hydroxide precipitation occurred in the pore space of the calcareous sand, consequently leading to a decrease in the permeability coefficient of the sand column [38,39], as depicted in Fig 7C. Synergistic effects emerged when both Ca^2^⁺ and Fe^3^ ⁺ were present: permeability decreased to 6.4 × 10 ⁻ ⁵ m·s ⁻ ¹ at 0.001 M Fe^3^⁺ and further declined to 1.6 × 10 ⁻ ⁵ m·s ⁻ ¹ at 0.03 M Fe^3^⁺ (Fig 7D). These values represent one- and two-order-of-magnitude reductions compared to conventional MICP-treated and untreated sand, respectively, demonstrating enhanced pore-clogging efficiency through combined biomineralization and physicochemical precipitation.

To more accurately describe the influence of ferric ion concentration on sand column reinforcement from a data perspective, we summarize the CaCO3 content, UCS and permeability coefficient of different experimental groups in Table 2.

**Table 2:** The influence of ferric ion concentration on the content of CaCO_3_, UCS and permeability coefficient of sand columns.

Parameters of sand columns	Ferric ion concentration (M)						
	0	0.001	0.003	0.005	0.01	0.02	0.03
**CaCO3 content (%)**	11.1	11.3	12.3	10.5	9.3	8.8	8.1
**UCS (kPa)**	185.5	399.1	2081.7	2535.1	2832.6	2256.4	1733.2
**Permeability coefficient (m/s)**	2.2 × 10^−4^	6.4 × 10^−5^	3.1 × 10^−5^	2.2 × 10^−5^	1.8 × 10^−5^	1.7 × 10^−5^	1.6 × 10^−5^

**Fig 8:**
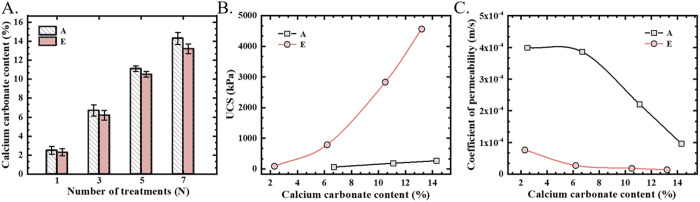
A) CaCO_3_ content versus number of treatments; B) UCS versus calcium carbonate content; C) permeability coefficient versus calcium carbonate content.

Comparative analysis of CaCO_3_ content, UCS, and permeability coefficient was conducted between control Group A and optimized Group E under different treatment cycles (Fig 8). The CaCO_3_ content of each group increased as the number of cementing solution treatments increased (Fig 8A). However, a significant divergence in CaCO_3_ content emerged between groups after three cycles of cementing treatment. This divergence can be attributed to the increased injection of Fe^3^⁺ , leading to a substantial decrease in the system's pH value, which in turn inhibited urease activity.

The mechanical properties of MICP-reinforced calcareous sand are closely related to the cementation cycle and the content of calcium carbonate. As the number of cementing solution treatments increases, the amount of CaCO_3_ precipitation gradually increases, improving the UCS. As shown in Fig 8B, after only one cementing solution treatment, the sand column of Group E has achieved effective cementation, with a UCS of 87.3 kPa; while Group A requires three cementing solution treatments with a UCS of 62 kPa. As the cementation level increases, the strength difference between the two groups gradually increases. After seven cementing solution treatments, the CaCO_3_ content of Group E is 13.2%, and the UCS is 4567.7 kPa; while under the same conditions, the CaCO_3_ content of Group A is 14.3%, and the UCS is only 267.5 kPa. It is evident that, under the same degree of CaCO_3_ precipitation, the UCS of the sand column in Group E (with added ferric ions) is approximately 17.1 times greater than that of Group A.

Fig 8C illustrates the relationship between the permeability coefficient and CaCO_3_ content for Groups A and E. After one cycle of cementing solution treatment, the CaCO_3_ content of Group A was 2.5%, and the permeability coefficient decreased from 2.6 × 10 ⁻ ^3^ m·s ⁻ ¹ of the untreated sand to 4 × 10 ⁻ ⁴ m·s ⁻ ¹, indicating that the MICP technology has a good pore-clogging ability. Under comparable CaCO_3_ content conditions, Group E achieved a permeability coefficient of 7.6 × 10 ⁻ ⁵ m·s ⁻ ¹, demonstrating a more excellent clogging effect, further confirming the synergistic pore-clogging effect of Fe^3^⁺ on MICP.

With the increase of treatment cycles, the permeability coefficient of Group A continued to decrease, reaching 9.6 × 10 ⁻ ⁵ m·s ⁻ ¹ after seven treatment cycles. In contrast, the permeability coefficient of Group E reached 1.35 × 10 ⁻ ⁵ m·s ⁻ ¹ after seven treatment cycles, which was two orders of magnitude lower than that of the untreated sand. This result indicates that the synergistic effect of ferric ions and MICP has broad application prospects in the field of seepage prevention.

### Microscopic testing and analysis

The MICP process involves bacteria decomposing urea and generating calcite crystals from Ca^2+^ ions. As Cui et al. [4,40] described, the newly formed calcite deposits and accumulates on the surface of existing crystals, eventually forming interconnected calcite clusters through oriented attachment. The reinforcement mechanism of sandy soil mainly includes three coupled processes: nucleation of calcite in soil pores, crystal accumulation, and subsequent crystal growth.

Biological calcium carbonate serves a dual function in soil improvement by causing both bio-clogging and bio-cementation [41]. Specifically, the content of the precipitate CaCO_3_, as a pore-filling material, is directly related to the treatment cycle and significantly affects the degree of soil porosity reduction. The efficiency of bio-clogging mainly depends on the total CaCO_3_ content, while the efficiency of bio-cementation is more dependent on the spatial distribution characteristics of CaCO_3_. Only bridge-like crystals formed between particles or precipitates at contact points—referred to as “effective crystals”—substantially contribute to enhancing soil strength.

**Fig 9:**
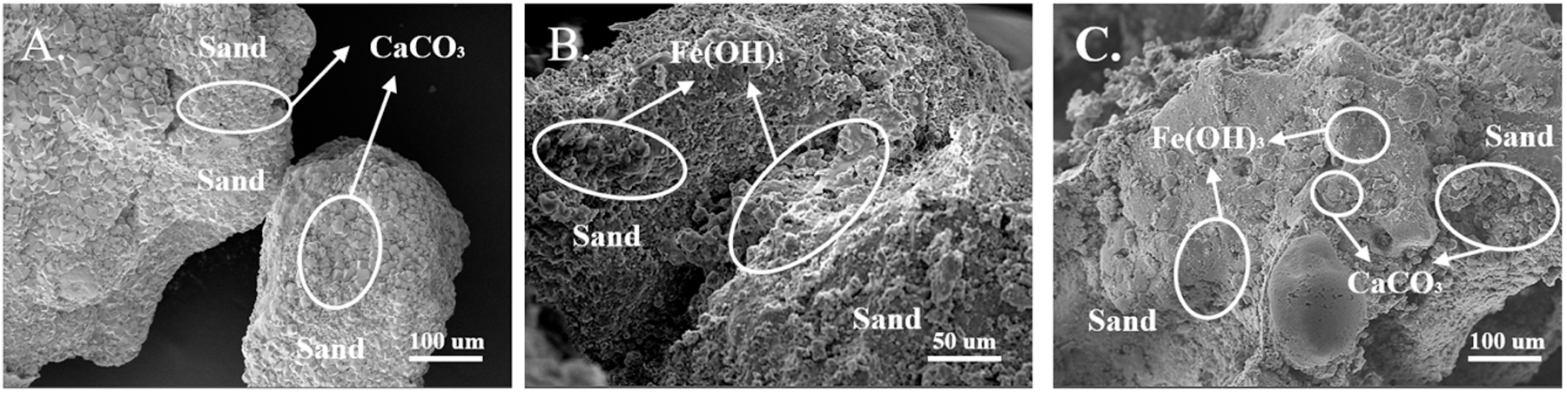
SEM images of sand column test. A) group A; B) group H; C) group **F.**

Fig 9A illustrates CaCO_3_ precipitation in MICP-reinforced calcareous sand. CaCO_3_ precipitates on the surface of calcareous sand grains, gradually encapsulating them as the degree of cementation increases. Furthermore, with the continued increase in calcium carbonate content, interparticle cementation occurs between calcium carbonate particles, resulting in the overall cementation of the sand column.

Introducing ferric ions into the cementing solution promotes the precipitation of ferric hydroxide, as illustrated in Fig 9B. In the absence of Ca^2+^, the reaction exclusively results in ferric hydroxide deposits. Microscopic analysis identifies two distinct precipitation patterns: ferric hydroxide either forms a coating on the particle surface or creates colloidal bridges between adjacent sand grains. As shown in Fig 6E, isolated ferric hydroxide precipitates fail to effectively cement sand particles and merely produce localized red aggregates on the sand particle surfaces.

In a co-precipitation system containing both calcium and ferric ions, synergistic crystallization occurs. The concurrent deposition of calcium carbonate and ferric hydroxide forms a continuous bonding layer at the particle interfaces, significantly enhancing the overall strength of the sand column, as demonstrated in Fig 9C.

**Fig 10:**
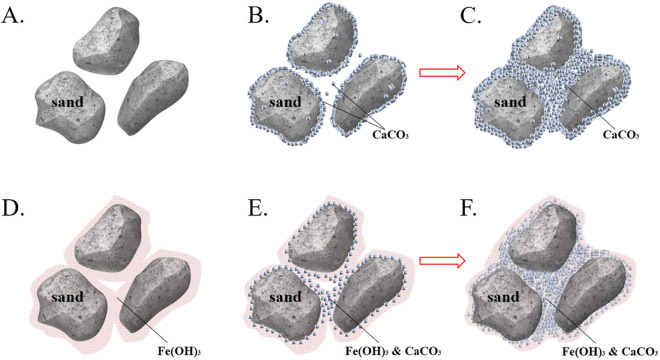
Mechanism demonstration diagrams. A) calcareous sand; **B)**-C) group A; D) group H; **E)**-F) group **F.**

Microstructure analysis reveals that MICP-reinforced calcareous sand exhibits distinct cementation patterns at different treatment stages (Fig 10A-C). During the initial stage of CaCO_3_ formation, crystals predominantly precipitate on the surface of sand particles (Fig 10A-B). To achieve significant enhancement in the strength of the sand column, repeated treatments with the cementing solution are required to establish a continuous and robust cementation network between sand grains. As multiple cementation cycles progress, the CaCO_3_ content progressively increases (Fig 10C), leading to pore clogging and reducing the permeability coefficient of the sand. In summary, while MICP effectively enhances UCS and reduces permeability, its practical application necessitates repeated cementation cycles, reflecting the inherent limitation of this technology in terms of crystal network formation efficiency.

In Group H, ferric ions undergo hydrolysis in the solution and form ferric hydroxide precipitates under ammonia conditions. These precipitates accumulate on the surface of sand grains and between adjacent particles (Fig 10D). This accumulation leads to pore clogging through the buildup of colloidal ferric hydroxide. Compared to untreated sand, the resulting bio-clogging effect significantly reduces the permeability coefficient of the sand; however, the lack of effective inter-particle bonding in ferric hydroxide precipitates limits their contribution to the mechanical strengthening of the sand matrix.

The MICP reaction and the hydrolysis of ferric ions exert distinct effects on pH levels. The hydrolysis of ferric ions releases H^+^, leading to a gradual acidification of the solution. When the concentration of ferric ions increases or repeated treatments are performed, the pH level drops markedly, thereby suppressing the precipitation efficiency of CaCO_3_. Within the pore network of sandy soil, ferric hydroxide deposition enhances inter-particle connectivity by forming cementation bonds between sand grains; meanwhile, calcium carbonate nucleates not only on the surface of sand particles but also on the interface of ferric hydroxide colloids. Stocks-Fischer et al. [42] observed via scanning electron microscopy that bacteria can act as nucleation sites during the mineralization process. With technological advancements, precise observation of microbial mineralization kinetics has become feasible. Wang [43] and Zambare [44] employed microfluidic analysis methods and provided definitive evidence for the bacterial-mediated nucleation mechanism through real-time interface monitoring.

Ferric hydroxide colloids form between sand grains, providing adhesion sites for bacteria and creating new nucleation sites for calcite precipitation. This fundamentally changes the distribution pattern of calcium carbonate within the sand pore space (as shown in Fig 10E), with ferric hydroxide facilitating the growth of calcite along the cementation interface. Consequently, effective inter-particle bonding can be achieved at a lower degree of cementation, thereby reducing the treatment cycles required and enhancing the reinforcement of calcareous sand. The precipitation behavior observed here may also reflect the substrate-controlled nucleation patterns reported by Janssen et al. [27], suggesting that substrate composition, in addition to solution chemistry, governs CaCO_3_ distribution and bonding efficiency. Additionally, the synergistic clogging effect of CaCO_3_-Fe(OH)_3_ co-precipitates further decreases the permeability coefficient of the sand.

As the number of treatments with the cementing solution increases, the CaCO_3_ content progressively rises (as shown in Fig 10F), strengthening the cementation network and significantly improving the UCS of the sand. The clogging effect of ferric hydroxide rapidly reduces the initial permeability of the sand, and its effect is superior to the traditional MICP clogging mechanism. However, during the ferric ion-assisted MICP reinforcement of calcareous sand, CaCO_3_ tends to preferentially deposit on existing precipitates. While this leads to a rapid decrease in permeability at the early stages, the reduction in permeability becomes increasingly limited as the cementation cycle progresses.

## Conclusions

In this study, a modified approach for MICP – reinforced calcareous sand is proposed, where the addition of ferric ion additives to the cementing solution can enhance the efficiency of MICP. The impacts of ferric ions on MICP – precipitated calcium carbonate, the UCS of the sand column, and the permeability coefficient were elucidated through an aqueous solution test, a sand column test, and a microscopic test. The specific findings are as follows:

(1) In the aqueous solution test, ferric ions increased the amount of calcium carbonate precipitated by MICP and changed the size and morphology of calcium carbonate crystals. Conversely, in the sand column test, when the concentration of ferric ions is relatively high or the injection of the cementing solution containing ferric ions is excessive, the amount of calcium carbonate precipitated decreases.
(2) In the sand column test, when ferric ions were added to the cementing solution, the sand column could be reinforced with single treatment of the cementing solution. After five injections, the UCS of the sand column could reach as high as 2832.63 kPa, whereas the UCS of the conventional MICP test group was merely 185.5 kPa.
(3) The hydrolysis of ferric ions was involved in the MICP reaction, resulting in the formation of Fe(OH)₃ - CaCO_3_ co – precipitation. This co-precipitation accelerated the reduction of the permeability coefficient of the sand column. After five treatments with the cementing solution, the permeability coefficient decreased by two orders of magnitude compared to that of the untreated sand.
(4) The mechanism underlying the reinforcing effect of ferric ions on the microbial mineralization of calcareous sands is the alteration of the distribution pattern of MICP – generated calcium carbonate within sandy soils. Ferric ions facilitate the formation of cementation surfaces between sand particles, which significantly enhances the strength of sandy soils and reduces the permeability coefficient.

## Supporting information

S1 Raw Data Raw data for statistical analysis and figure generation. (PDF)
