# Supplementary material for: Enhancing microbial-induced calcium carbonate precipitation efficiency in calcareous sands through ferric ion additives: A comprehensive experimental investigation
Source: PLoS One. 2025 Jul 9;20(7):e0327568. doi: 10.1371/journal.pone.0327568 (PMC12240300; doi:10.1371/journal.pone.0327568)
Supplement: S1 Raw Data — (PDF) [file pone.0327568.s001.pdf]

Fig 4A

	Ferric ion concentration (M)					
	0	0.001	0.003	0.005	0.01	0.02
Statistical values of $CaCO_3$ particle size (um)	14	6	28	27	25	30
	3	3	20	45	17	10
	2	12	30	25	45	5
	4	4	27	30	28	8
	5	3	25	42	30	12
	2	5	30	30	25	30
	5	5	33	40	28	15
	2	5	26	28	35	7
	4	11	22	48	25	27
	2	3	28	33	40	6
	7	5	25	30	17	13
	3	7	32	50	28	10
	4	3	30	45	30	8
	15	11	28	36	25	25
	5	4	26	50	44	28
	2	5	29	28	30	
	3	7	31	35	28	
	5	4	27	50	35	
	7	11	28	32	40	
	3	5	27	28	25	
	6	12	29	30		
	4	3	30	48		
	13	4	33	50		
	11		33	35		
	14		28	50		
Average value (um)	5.8	6	28.2	37.8	30	15.6
Maximum value (um)	15	12	33	50	45	30
Minimum value (um）	2	3	20	25	17	5

Fig 5A

Ferric ion concentration (M)	Parallel sample data (g)			Average value (g)	Standard deviation
	Sample 1	Sample 2	Sample 3		
0	6.75	6.56	6.04	6.45	0.3
0.001	8.12	6.94	7.8	7.62	0.5
0.003	11.07	11.06	9.58	10.57	0.7
0.005	11.32	12.34	12.43	12.03	0.5
0.01	12.95	13.95	14.05	13.65	0.5
0.02	12.85	11.85	12.95	12.55	0.5
0.03	7.91	7.93	7.29	7.71	0.3

Fig 5B

Time (h)	pH changes in different experimental groups			
	Bacteria+Urea	Bacteria+Urea+Fe^{3+}	Bacteria+Urea+Ca^{2+}	Bacteria+Urea+Ca2++Fe^{3+}
0	8.57	2.32	6.67	2.1
1	9.2	4	7.1	2.38
2	9.65	5.1	7.13	2.68
4	9.8	7.22	7.16	3.3
6	9.86	7.92	7.16	3.8
9	9.88	8.8	7.17	4.2
12	9.88	9.26	7.18	4.68
18	9.89	9.46	7.19	5.6
24	9.9	9.64	7.2	6.6

Fig 5C

Time	The influence of different concentrations of ferric ions on pH						
	0	0.001	0.003	0.005	0.01	0.02	0.03
0	6.67	6.27	3.43	2.74	2.39	2.1	2.06
1	7.1	6.85	6.48	5.12	3.13	2.38	2.34
2	7.13	6.94	6.75	6.3	4.7	2.68	2.45
4	7.16	7.05	6.9	6.6	6	3.3	2.82
6	7.16	7.11	7.09	6.72	6.35	3.8	3.15
9	7.17	7.15	7.09	6.8	6.58	4.2	3.62
12	7.18	7.17	7.1	6.87	6.58	4.68	4.28
18	7.19	7.17	7.1	6.9	6.65	5.6	5.2
24	7.2	7.18	7.2	6.92	6.75	6.6	6.37

Fig 7A

Ferric ion concentration (M)	Parallel sample data (%)			Average value (%)	Standard deviation
	Sample 1	Sample 2	Sample 3		
0	11.32	10.68	11.3	11.1	0.3
0.001	11.8	11.48	10.62	11.3	0.5
0.003	12.8	12.65	11.45	12.3	0.6
0.005	10.8	10.09	10.61	10.5	0.3
0.01	9.66	8.74	9.5	9.3	0.4
0.02	9.01	8.87	8.53	8.8	0.2
0.03	8.5	8.41	7.4	8.1	0.5

Fig 7B

Ferric ion concentration (M)	Parallel sample data (kPa)			Average value (kPa)	Standard deviation
	Sample 1	Sample 2	Sample 3		
0	111.71	205.24	239.47	185.47	54
0.001	465.34	423.37	308.71	399.14	66.2
0.003	1989.51	2106.42	2149.21	2081.71	67.5
0.005	2670.05	2584.46	2350.64	2535.05	135
0.01	2954.63	2877.29	2665.98	2832.63	122
0.02	2375.35	2299.91	2093.8	2256.35	119
0.03	1839.17	1771.97	1588.37	1733.17	106

Fig 7C

Experimental group	Parallel sample data (m/s)			Average value (m/s)	Standard deviation
	Sample 1	Sample 2	Sample 3		
Untreated sand	0.00269	0.00261	0.00247	0.0026	9.00E-05
A	2.70E-04	2.38E-04	1.52E-04	2.20E-04	5.00E-05
H	6.90E-04	6.52E-04	5.48E-04	6.30E-04	6.00E-05

Fig 7D

Ferric ion concentration (M)	Parallel sample data (m/s)			Average value (m/s)	Standard deviation
	Sample 1	Sample 2	Sample 3		
0.001	6.70E-05	5.99E-05	6.51E-05	6.40E-05	3.00E-06
0.003	3.35E-05	3.19E-05	2.76E-05	3.10E-05	2.50E-06
0.005	2.63E-05	2.35E-05	1.61E-05	2.20E-05	4.30E-06
0.01	2.12E-05	1.91E-05	1.36E-05	1.80E-05	3.20E-06
0.02	1.97E-05	1.80E-05	1.33E-05	1.70E-05	2.70E-06
0.03	1.94E-05	1.73E-05	1.14E-05	1.60E-05	3.40E-06

Fig 8A

Number of treatments (N)	Parallel sample data (%) group A			Average value (%)	Standard deviation	Parallel sample data (%) group E			Average value (%)	Standard deviation
	Sample 1	Sample 2	Sample 3			Sample 1	Sample 2	Sample 3		
1	2.9	2.65	1.95	2.5	0.4	2.68	2.44	1.78	2.3	0.38
3	7.3	6.92	5.88	6.7	0.6	6.7	6.38	5.52	6.2	0.5
5	11.4	11.21	10.69	11.1	0.3	10.8	10.61	10.09	10.5	0.3
7	14.9	14.52	13.48	14.3	0.6	13.7	13.38	12.52	13.2	0.5

Fig 8B

CaCO3 content (%) Group A	UCS (kPa)	CaCO3 content (%) Group E	UCS (kPa)
2.5	0	2.5	87.3
6.7	62	6.7	787.9
11.1	185.47	11.1	2832.63
14.3	267.5	14.3	4567.7

Fig 8C

CaCO3 content (%) Group A	Permeability coefficient (m/s)	CaCO3 content (%) Group E	Permeability coefficient (m/s)
2.5	3.99E-04	2.5	7.60E-05
6.7	3.86E-04	6.7	2.70E-05
11.1	2.20E-04	11.1	1.80E-05
14.3	9.58E-05	14.3	1.35E-05
